# Absence of CX3CR1 impairs the internalization of Tau by microglia

**DOI:** 10.1186/s13024-017-0200-1

**Published:** 2017-08-15

**Authors:** Marta Bolós, María Llorens-Martín, Juan Ramón Perea, Jerónimo Jurado-Arjona, Alberto Rábano, Félix Hernández, Jesús Avila

**Affiliations:** 1 Centro de Investigación Biomédica en Red de Enfermedades Neurodegenerativas (CIBERNED), Valderrebollo 5, 28041 Madrid, Spain; 2 Centro de Biología Molecular “Severo Ochoa” CSIC-UAM, Universidad Autónoma de Madrid, C/ Nicolás Cabrera 1, 28049 Madrid, Spain; 3 Department for Molecular Biology, Faculty of Sciences, Universidad Autónoma de Madrid, Madrid, Spain; 4 Neuropathology Department, CIEN Foundation, Madrid, Spain

**Keywords:** Tau, Alzheimer’s disease, Tauopathies, CX3CR1, Phagocytosis, Microglia

## Abstract

**Background:**

Extracellular Tau is toxic for neighboring cells, and it contributes to the progression of AD. The CX3CL1/CX3CR1 axis is an important neuron/microglia communication mechanism.

**Methods:**

We studied Tau clearance by microglia both in vitro (microglia primary cultures treated with Cy5-Tau, affinity chromatography to study the binding of Tau to CX3CR1, and Tau-CX3CL1 competition assays) and in vivo (stereotaxic injection of Cy5-Tau into WT and CX3CR1^−/−^ mice). The expression of CX3CR1, CX3CL1 and the microglial phagocytic phenotype were studied in brain tissue samples from AD patients.

**Results:**

Tau binding to CX3CR1 triggers the internalization of the former by microglia, whereas S396 Tau phosphorylation decreases the binding affinity of this protein to CX3CR1. Of note, the progressive increase in the levels of phosho-Tau occurred in parallel with an increase in CX3CR1. In addition, our studies suggest that the phagocytic capacity of microglia in brain tissue samples from AD patients is decreased. Furthermore, the CX3CR1/CX3CL1 axis may be impaired in late stages of the disease.

**Conclusions:**

Our data suggest that the CX3CR1/CX3CL1 axis plays a key role in the phagocytosis of Tau by microglia in vitro and in vivo and that it is affected as AD progresses. Taken together, our results reveal CX3CR1 as a novel target for the clearance of extracellular Tau.

**Electronic supplementary material:**

The online version of this article (doi:10.1186/s13024-017-0200-1) contains supplementary material, which is available to authorized users.

## Background

Tau is a microtubule-associated protein that plays a critical role in the pathogenesis of several related disorders collectively known as tauopathies [1]. Alzheimer disease (AD) is the most prevalent tauopathy and the main cause of dementia among older adults. In this disease, intracellular Tau forms filamentous structures of aggregated and hyperphosphorylated protein (phospho-Tau), which are associated with neuronal death. Tau is released into the extracellular space through physiological and pathological mechanisms and can be toxic for neighboring cells. This effect may contribute to the progression of a number of neurodegenerative diseases [2, 3].

Microglia are the resident immune cells of the central nervous system (CNS) [4, 5]. Under physiological conditions, they are quiescent and scattered throughout the CNS [6]. In response to CNS damage, microglia become activated to exert their classic role as “scavengers”. They proliferate, change their morphology, and phagocytose cells and structures that may impair CNS homeostasis [7]. These microglial functions are regulated by communication between neurons, astrocytes and microglia [8] through cytokines, chemokines, trophic factors, and other molecules.

One signaling pathway through which neurons and microglia communicate is the neuronal cytokine CX3CL1 (fractalkine) and its microglial receptor (CX3CR1) [9]. CX3CL1 is expressed by neurons either as a membrane-bound or a secreted ligand [10, 11]. The binding of CX3CL1 to CX3CR1 maintains microglia in an “off” state [12], thereby inhibiting the release of pro-inflammatory cytokines [13, 14]. In contrast, deficiency of CX3CL1 or CX3CR1 leads to an increased production of pro-inflammatory molecules [15]. While there is a correlative link between neuroinflammation and tauopathies and several studies have shown that microglia drive Tau pathology and contribute to the spread of pathological Tau [16, 17], there is little mechanistic evidence that altered microglial activity is involved in the pathogenesis of these diseases [18–20].

Our previous studies, among others, have demonstrated that extracellular Tau can be taken up by microglia [21, 22]; however, the precise mechanisms involved in this process are unknown. To study these mechanisms, here we used mice in which *Cx3cr1* was replaced with a cDNA encoding enhanced green fluorescent protein (EGFP) (CX3CR1^−/−^ mice) [23]. Our results indicate that, in the absence of CX3CR1, microglia had an altered phagocytic response to Tau. In particular, CX3CR1 deficiency was associated with impaired uptake and degradation of Tau by microglia both in vitro and in vivo. Furthermore, our study is the first to demonstrate that Tau binds to CX3CR1, that by doing so Tau increases its own internalization by microglia, and that Tau competes with the natural ligand of CX3CR1, namely CX3CL1, to bind to this receptor. These data, together with the observation that patients with an advanced stage of AD show an increase in the expression of the CX3CL1/CX3CR1 axis in the hippocampus, identify CX3CR1 as a receptor that plays a pivotal role in the regulation of Tau phagocytosis by microglia.

## Methods

### Peptides

The recombinant human Tau isoform containing 2 N-terminal inserts and 4 microtubule binding repeats (Tau42 [24]) was isolated as described previously [25]. Purified Tau42, 1 mg/ml, was used for affinity chromatography assays or was labeled with sulfoindocyanine Cy5 dye (GE Healthcare, UK), as described previously [21] following the manufacturer’s recommendations. As a control for Tau specificity, Bovine serum albumin (BSA) (1 mg/ml), was labeled with Cy5 dye following the same protocol. The synthetic CX3CR1 peptide sequence: DQFPESVTENFEYDDLAEAC (aa 2–21) containing the binding region to CX3CL1, and synthetic c-terminal Tau peptide sequence: HLSNVSSTGSIDMVDSPQLATLADEV (aa 407–432), containing the C-terminal fragment of Tau with negatively charge amino acids like D and E, were purchased from Abyntek. Full-length CX3CL1 peptide was obtained from R&D Systems, USA.

### Animals

CX3CR1 (KO) mice with a targeted mutation (B6.129P–Cx3cr1tm1Litt/J) were obtained from the Jackson Laboratories (Bar Harbor, Maine). Two-month-old KO and control C57Bl/6 (WT) (Harlan Laboratories, Netherlands) mice were used for the in vivo experiments involving stereotaxic injection of 1 mg/ml Tau-Cy5 or PBS-Cy5. Mice were housed in a specific pathogen-free colony facility, in accordance with European Community Guidelines (directive 86/609/EEC), and handled following European and local animal care protocols. Animal experiments received the approval of the CBMSO’s Ethics Committee and the National Ethics Committee (AEEC-CBMSO-62/14).

### Primary cultures

Microglia were cultured from the cerebral cortices of 2-day-old mice, as described previously [21]. These cells were seeded onto 24-well plates (3 × 10^5^ cells/well) with coverslips. Adherent cells were incubated for 48 h before being used for the experiments.

### Human subjects

The use of tissue samples from the human hippocampus was coordinated by the local Brain Bank (Banco de Tejidos CIEN Foundation, Madrid), following national laws and international ethical and technical guidelines on the use of human samples for biomedical research purposes. In all cases, brain tissue donation, processing, and use for research purposes were in compliance with published protocols (Best Practices for Repositories [26]), which include informed consent for brain tissue donation from living donors and the approval of the whole donation process by an Ethics Committee. Epidemiological information on the subjects involved in this study is included in Table 1. Hippocampal samples were obtained post-mortem and were post-fixed O/N in 4% PFA.

**Table 1:** Table shows epidemiological information on human brain tissue samples from the hippocampus

	CODE	AGE	GENDER	POST-MORTEM INTERVAL	BRAAK-TAU	DIAGNOSIS
CONTROL	1	65	MALE	3	**0**	Methastasis
	2	52	FEMALE	6	**0**	Mental retardation
	3	75	FEMALE	1	**0**	Neuropathy
	4	71	MALE	5,5	**0**	FTD
	5	78	MALE	4	**0**	Dyslipidemia
	6	80	MALE	3	**0**	Neuropathy
	7	43	MALE	5	**0**	Leukaemia
	8	56	FEMALE	5	**0**	ALS
	9	68	MALE	4	**0**	MSA
	10	41	MALE	6	**0**	ALS
BRAAK-TAU I and II	11		FEMALE	6	**I**	ALS
	12	67	FEMALE	6	**I**	ALS
	13	82	MALE	5	**I**	PD
	14	73	MALE	9	**I**	AD
	15	76	MALE	3	**II**	PD
	16	73	MALE	6,5	**II**	Vascular dementia
BRAAK-TAU III and IV	17	80	FEMALE	5	**III**	ALS
	18	85	MALE	8,5	**III**	Dementia
	19	70	FEMALE	9	**III**	ALS
	20	82	FEMALE	3,5	**IV**	FTD
	21	79	MALE	4,5	**IV**	AD
	22	62	MALE	5	**IV**	PD
BRAAK-TAU V and VI	23	93	FEMALE	5,5	**V**	AD
	24	92	MALE	4	**V**	AD
	25	73	MALE	5	**V**	AD
	26	80	MALE	5	**V**	AD
	27	81	MALE	2,5	**V**	AD
	28		FEMALE	4	**V**	AD
	29	85	MALE	2,5	**V**	AD
	30	89	FEMALE	6	**V**	AD
	31	93	MALE	8	**V**	AD
	32	86	FEMALE	?	**V**	AD
	33	82	FEMALE	?	**V**	AD
	34	78	FEMALE	6,5	**V**	AD
	35	84	MALE	4	**VI**	AD
	36	91	FEMALE	3	**VI**	AD
	37	92	FEMALE	?	**VI**	AD
	38	83	FEMALE	3,5	**VI**	AD
	39	87	MALE	10	**VI**	AD
	40	90	FEMALE	10	**VI**	AD
	41	89	FEMALE	4,5	**VI**	AD
	42	60	MALE	4,5	**VI**	AD
	43	55	MALE	5	**VI**	AD
	44	92	MALE	6	**VI**	AD
	45	77	MALE	9	**VI**	AD

Control, Braak-Tau I and II, Braak-Tau III and IV, and Braak-Tau V and VI groups are shown. Information regarding code, age, gender, postmortem interval, Braak-Tau stage and diagnosis of each sample is indicated

### Tau internalization assay

Primary microglia cultures were treated with either Tau-Cy5, BSA-Cy5 or vehicle (phosphate saline buffer (PBS)-Cy5) at 1 μM for 0, 5, 30 and 60 min. In addition, microglia cultures were treated with phospho-TauCy5 or control Tau-Cy5 at 1 μM for 30 min. For the competition assay, microglia cultures were treated with CX3CL1 (5 ng / ml) for 30 min. Afterwards, 1 μM Tau-Cy5 was added to the culture for 5 min. After treatment, cells were washed three times with PBS in order to remove the excess Tau attached to the membrane. Cells were fixed with 4% paraformaldehyde in 0.1 N phosphate buffer (PB) for the immunofluorescence analysis.

### Tau membrane binding assay

Primary microglia cultures were plated and after 48 h were treated with 1 μM Tau-Cy5 at for 5 min at 4 °C. These experimental conditions allow the binding of Tau to the surface of the cells, thereby minimizing the internalization of Tau into microglia. After treatment, cells were washed only once with PBS. Cells were fixed with 4% paraformaldehyde in 0.1 N PB, and Tau bound to microglia membrane surface was measured by immunofluorescence analysis.

### Microglia activation assay

Primary microglia cultures were treated with Tau-Cy5 or vehicle PBS-Cy5 at 1 μM for 5 and 30 min. In order to avoid the activation of the cells caused by the addition of the new medium, the first time represented is 5 min after treatment. Cells were washed three times with PBS after treatment in order to remove the excess Tau attached to the membrane. They were then fixed with 4% paraformaldehyde in PB for the immunofluorescence analysis of microglia activity using CD68 marker.

### Immunocytochemistry

After treatment, cells were fixed in 4% paraformaldehyde in PBS. Microglia were then stained with rabbit anti-Iba1 (1:1000, Wako, Japan), mouse anti-Cy5 (1:2000, GE Healthcare, UK) and rat anti-CD68 (1:1000, Abcam, UK), all diluted in 1% bovine serum plus 1% Triton in 0.1 N PB. For Tau membrane binding assays, antibodies were diluted in the same buffer without Triton. Cells were then incubated with a donkey anti-goat IgG conjugated to Alexa Fluor 488, donkey anti-rat IgG conjugated to Alexa Fluor 555, donkey anti-mouse IgG conjugated to Alexa Fluor 647 (1:1000), and DAPI (Merck, USA) at 1:10,000 dilution.

### Stereotaxic surgery and sacrifice

Mice were anesthetized with Isoflurane and placed in a stereotaxic frame. Coordinates (mm) relative to the Bregma in the anteroposterior, mediolateral, and dorsoventral planes were as follows: dentate gyrus (DG) of the hippocampus [− 2.0; ±1.4; −2.2]. Next, 2 μl/DG of Tau-Cy5 (1 mg/ml) or PBS-Cy5 solution was infused at 0.2 μl/min via a glass micropipette. After 1 and 8 weeks, mice were fully anesthetized by means of an intraperitoneal injection of 50 μl of pentobarbital and transcardially perfused with 0.9% saline followed by 4% paraformaldehyde in PB. Brains were removed and post-fixed overnight in the same fixative.

### Immunohistochemistry

Sagittal murine brain sections were obtained on a Leica VT1200S vibratome (50-μm thick sections). Immunohistochemistry was performed as described previously [27]. For all the analyses, series of brain slices were made up randomly of one section from every ninth. Whole series, containing 8–10 sections, were used for the immunohistochemical detection of Iba1, CX3CR, CX3CL1 and Tau. For mice, the mouse anti-Cy5 (1:2000, GE Healthcare, UK) and rabbit anti-Iba1 (1:500, Wako, Japan) antibodies were used, both in 1% (*v*/v) bovine serum plus 1% (*v*/v) triton in 1 N PB. For human tissue, the rabbit anti-phospho-Tau S396 (p-S396) (1:500, Life Technologies, USA), goat anti-Iba1 (1:500, Abcam, UK), goat anti- CX3CR1 (1:500, Sigma, USA) and goat anti-CX3CL1 (1:500, R&D, USA) antibodies were used, all in 1% (*v*/v) bovine serum plus 1% (*v*/v) Triton in 1 N PB. Primary antibodies were incubated for 48 h at 4 °C. The following secondary Alexa-conjugated antibodies were used at a final concentration of 1:1000 diluted in 1% (*v*/v) bovine serum plus 1% (*v*/v) triton in 1 N PB: donkey anti-mouse IgG conjugated to Alexa Fluor 555 or 647; donkey anti-rabbit IgG conjugated to Alexa Fluor 488 or 555; and donkey anti-goat IgG conjugated to Alexa Fluor 647 (Thermo Fisher,USA). Secondary antibodies were incubated overnight at 4 °C. All sections were counterstained with DAPI at 1:5000 dilution. For human slices, immunohistochemistry was performed as described, adding an autofluorescence eliminator reagent (Millipore, USA) after DAPI incubation in order to remove any autofluorescence of the tissue [28].

### Confocal image acquisition and image analysis

Confocal stacks of images (Z-axis interval: 1 μm) were obtained in a Zeiss LSM710 confocal microscope under either 25X, 40X or 63X oil objectives. Images were analyzed using ImageJ Version 1.46r (National Institutes of Health, Bethesda, MD). The JACoP plugin [29] was used for colocalization analysis.

- Fluorescence intensity analysis: The fluorescence intensity of Cy5, Iba1, phospho-tau (p-396), CX3CR1 and CX3CL1 was determined in vivo. In order to measure the fluorescence intensity of Cy5 staining, three confocal images per mouse were obtained under a 40X magnification objective in the hippocampal DG. Images were subjected to a fixed threshold, and the area above the threshold that was Cy5^+^ was measured. Data relative to fluorescence intensity are thus presented as % of positive area. To measure phospho-tau (p-S396) fluorescence intensity in human tissue, three-to six images per subject were obtained in the hippocampal region. Images were subjected to a fixed threshold and the area above the threshold that was p-S396^+^ was measured. Data relative to fluorescence intensity are thus presented as % of positive area. In order to analyze the fluorescence intensity of CX3CR1 and CX3CL1 staining, the area of the granule layer (GL) of the dentate gyrus (DG) was drawn on 25X confocal images. Images were subjected to a fixed threshold and the area above the threshold that was CX3CR1^+^ or CX3CL1^+^ was measured and then divided by the total area of the GL. Data relative to fluorescence intensity are thus presented as % of positive area. Three-to-six images were obtained per mouse or subject.

- Colocalization analysis: The colocalization between Cy5 and Iba1 was measured in vitro and in vivo (in the brains of stereotaxically injected mice). The colocalization between CD68 and Iba1 was measured in vitro. For the in vitro measurements, internalized Tau-Cy5 or Tau-Cy5 attached to the membrane surface and CD68 expression were measured by quantifying the levels of Cy5 or CD68 staining inside, or levels of Cy5 on the surface of microglial cells. For this purpose, the contour of individual microglial cells was drawn in the Iba1 channel and the total area was measured on 40X magnification images. Images were subjected to an invariant threshold and the Cy5^+^ or CD68^+^ area above the threshold was measured and then divided by the selected area of the microglial cell. Colocalization is presented as % of Cy5^+^ or % of CD68^+^ positive areas. 150 cells were analyzed for each experimental condition and time point. In the case of the in vivo colocalization analysis between Iba1 and either Cy5 or phospho-Tau staining, three confocal stacks of images were obtained under a 40X magnification objective in the DG per mouse or per human tissue, respectively. Images were subjected to an invariant threshold for each of the channels (Cy5 and Iba1), and Mander’s coefficients were calculated.

- Quantifications relative to microglial cells in human brain. The number of microglial cells was quantified as previously described [28]. Briefly, microglial (Iba1^+^) cells were counted under a LSM710 Zeiss confocal microscope (63× Oil immersion objective) using the physical dissector method adapted for confocal microscopy [28]. Stacks of images (Z-interval: 1 μm) were obtained and the number of microglial cells was calculated and then divided by the reference volume of the stack. Cell counts are presented as cell density (number of cells/μm3). The area of the nucleus was measured in the image in which the nucleus showed the maximal profile using ImageJ software. The number of phagocytic pouches [30] per cell was quantified in all the cells present in each stack of images, as previously described [31]. A minimum of 100 cells per patient were analyzed. Merged images of blue (DAPI) and green (Iba1) channels were used to distinguish between the nucleus (DAPI staining was prominent) and the phagocytic pouches (DAPI staining was absent) of microglial cells.

### Phosphorylation of tau by GSK-3β

Tau42 and Tau-Cy5 was phosphorylated by GSK-3β (Sigma, USA) in a buffer containing 25 mM MOPS pH = 7.2; 12.5 mM glycerol-2-phosphate; 25 mM MgCl2; 5 mM EGTA; 2 mM EDTA; 0.25 DTT and 5.5 mg of ATP for 60 min at 30 °C, followed by 5 min at 100 °C. The reaction was centrifuged at 14,000 rpm for 10 min at 4 °C, and the supernatant containing phospho-Tau was collected. The control of the reaction was performed following the same protocol without GSK-3β. Tau phosphorylation was corroborated by western blot.

### Affinity chromatography

This method was used to assay the binding between CX3CR1 and Tau. Affinity chromatography was performed using the protocol described previously [32] with some modifications as follows: CNBr-activated Sepharose 4B was purchased from Sigma (USA). The medium was prepared following the manufacturer’s instructions. One mg of the synthetic peptide containing the CX3CR1 binding site was mixed with 0.3 g of CNBr-activated Sepharose 4B in a buffer which contained 0.1 M-NaHCO3, pH 8.5, and 0.5 M-NaCl. The mixture was incubated and gently agitated for 1.5 h at RT, and the coupling of the peptide to the resin was stopped by the addition of 0.1 M-Tris, pH 8.0. The resin was washed and equilibrated with buffer A (0.1 M-Mes (pH 6.4)/0.5 mM-MgCl2/2 mM-EGTA). One mg of Tau42 protein dialyzed in buffer A was added to the column. The exclusion volume was collected in aliquots and analyzed by immunoblotting. The resin was washed and equilibrated again with buffer A in order to remove the excess Tau42 that was not bound. The protein bound to the column was eluted by addition of 0.5 M-NaCl in buffer A and collected in 250-μl aliquots for analysis. The same protocol was used to test the binding of CX3CL1 (positive control), ct-Tau (negative control) and phospho-Tau to the CX3CR1 peptide bound to the resin. The elution volume for each peptide was analyzed by immunoblotting.

To study whether Tau and CX3CL1 compete for binding to CX3CR1, a competitive assay was performed using the same protocol as for affinity chromatography. In this case, Tau (control) or Tau plus CX3CL1 were added to the column. The elution volume was analyzed by immunoblotting.

### Immunoblotting

The phosphorylation of Tau by GSK3β was determined by western blotting. An extract of Tau42 was used as a control. Tau42 without GSK3β was used as a control of the reaction. Proteins were then separated on 10% sodium dodecyl sulfate-polyacrylamide gels before being transferred electrophoretically onto polyvinylidene difluoride membranes (GE Healthcare, UK). The membranes were blocked for 2 h with 2% (*w*/*v*) skimmed milk powder in 50 mm Tris-buffered saline, pH 8, containing 0.05% (*v*/v) Tween 20 (TBS-Tween), and incubated overnight at 4 °C with rabbit anti-phospho-Tau (p-S396) antibody (1:1000 dilution). Protein expression was detected using HRP-conjugated secondary antibody (1:10,000 dilution). For quantification of immunoreactivity, images of blots were analyzed using ImageJ Version 1.46r (National Institutes of Health, Bethesda, MD).

Aliquots obtained from the affinity chromatography were dotted onto nitrocellulose sheets, blocked with 0.05% Tween-20 in phosphate-buffered saline (PBS), and incubated with mouse anti-Tau5 (Calbiochem, USA), goat anti-CX3CL1 (R&D, USA), mouse anti-Tau46 (Abcam,UK) or rabbit anti-phospho-Tau (p-S396) (Life Technologies, USA) antibodies (1:1000 dilution in PBS, containing 0.05% Tween-20) for 2 h at RT. Protein expression was detected using HRP-conjugated secondary antibodies (1:10,000 dilution). To measure immunoreactivity, images of blots were analyzed using ImageJ Version 1.46r (National Institutes of Health, Bethesda, MD). The results were presented as the percentage of Tau5 (Tau42), CX3CL1, Tau46 (ct-Tau) and p-S396 (phospho-Tau) in each fraction relative to the total exclusion volume.

### Statistical analysis

Statistical analysis was performed with GraphPad Prism software, Version 5.01. Data were tested by one-way ANOVA. Post-hoc comparisons were analyzed using Tukey’s test. Differences were considered statistically significant when the probability, p, of the null hypothesis was ≤0.05. Data are presented as the means ± S.E. All in vitro results were obtained in at least three independent experiments.

## Results

### The absence of CX3CR1 specifically decreases the uptake of tau by microglia

**Fig. 1:**
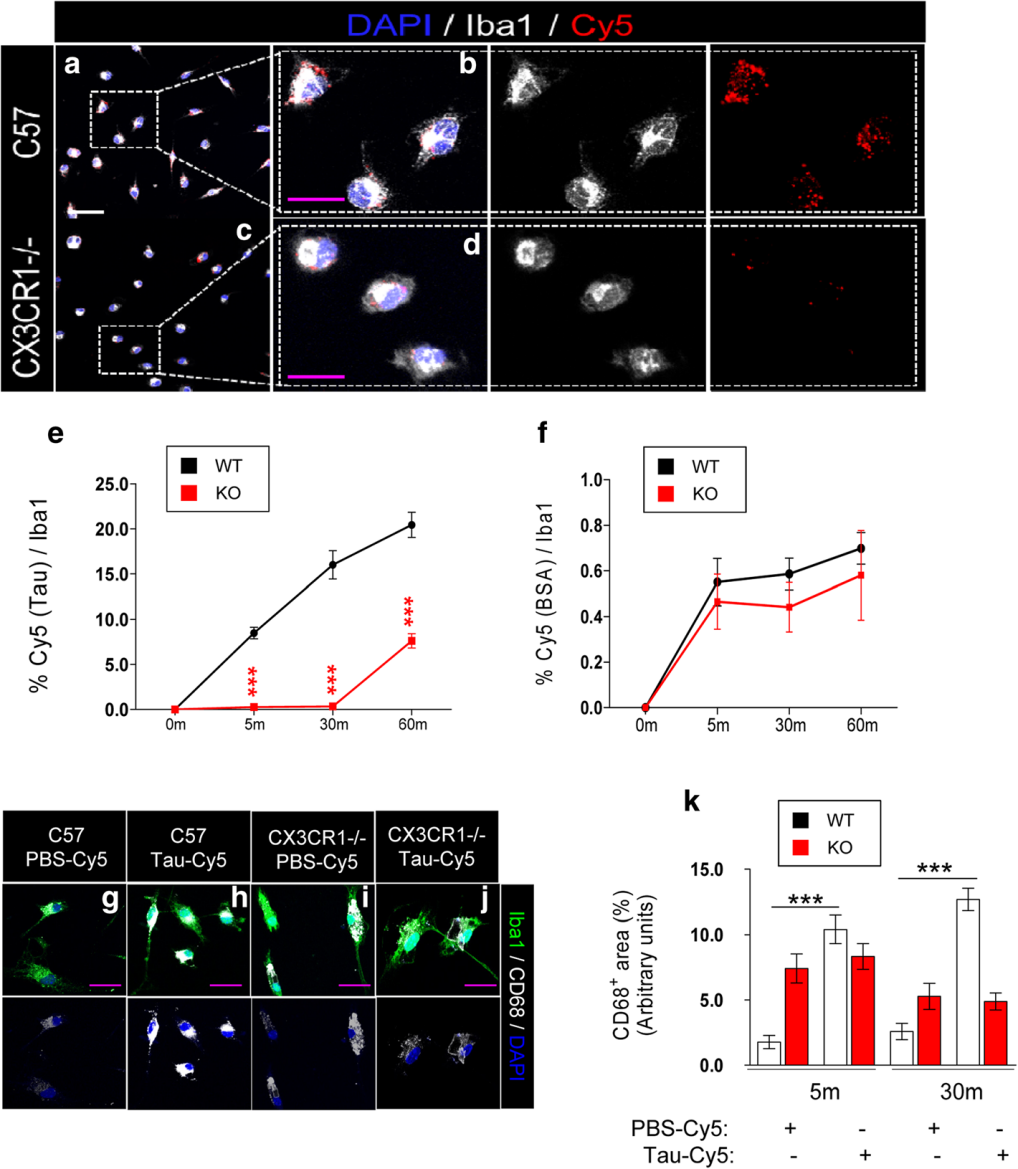
The absence of CX3CR1 leads to a decrease in extracellular Tau internalization by microglia in vitro. Representative immunofluorescence images of primary cultures of microglia derived from WT mice (**a**) and their high-power magnification (**b**), and from KO animals (**c**) and their magnification image (**d**). Cells were incubated with 1 μM Tau-Cy5. Immunofluorescence images for Iba1 (*white*) and Cy5 (*red*) are shown separately. (**e**) Quantification of Cy5^+^ area in individual microglial cells treated with either Tau-Cy5, or BSA-Cy5 (f) for 0, 5, 30 and 60 min. **g**-**l** Representative images of primary microglia cultures from WT mice treated with PBS-Cy5 (**g**) or Tau-Cy5 (**h**) and from KO animals treated with PBS-Cy5 (i) or Tau-Cy5 (**j**) showing CD68 (*white*) and Iba1 (*green*) staining. **k** Quantification of CD68^+^ area in individual WT and KO microglia after treatment with PBS or Tau at 0 and 30 min. Bars show means ± S.E. ∗∗∗*p* ≤ 0.001. White scale bar: 100 μm, purple scale bar: 50 μm

In our previous work, we showed the temporary course of Tau uptake by wildtype microglia [21]. Following the same protocol, primary microglia cultures derived from C57 (WT) and CX3CR1−/− (KO) mice were incubated with Tau42 labeled with Cy5 (Tau-Cy5), or control PBS-Cy5 at 1 μM of final concentration for 0, 5, 30 and 60 min. After this time, the presence of internalized Tau was calculated as percentage of Cy5^+^ area inside the microglial cell. Tau-Cy5 (red) was detected, for the first time, inside microglia (white) of WT mice (Fig. 1a–e) after 5 min of incubation. However, the Cy5 signal was absent in KO microglia at the same time point and only became observable after 60 min of treatment. However, this signal was reduced in comparison to WT microglia at this time point (Fig. 1c–e).

In order to elucidate whether the effect of CX3CR1 deletion on the phagocytosis of Tau is specific or whether it impairs general microglial phagocytic capacity, the internalization of BSA-Cy5 was studied as a control. Following the same protocol, primary microglia cultures derived from WT and KO mice were incubated with 1 μM BSA-Cy5 for 0, 5, 30 and 60 min. After this time, internalized BSA was calculated as percentage of Cy5^+^ area inside the microglial cell (Fig. 1f). There were no differences in the amount of BSA detected in cells at any time tested. The results indicated that CX3CR1 deletion did not affect the general phagocytic capacity of microglia and further confirmed that CX3CR1 is a specific receptor involved in Tau phagocytosis by microglia.

### Microglia activation is impaired in KO mice

Since the activation of microglia increases the phagocytosis of these cells [7] and we observed an impairment of Tau internalization by KO microglia, we studied whether the microglia derived from WT and KO mice are activated equally by the presence of Tau. In this regard, the CD68 signal was used as a measurement of microglial activation [30, 33]. WT (Fig. 1g and h) and KO (Fig. 1i and j) microglia were incubated with PBS-Cy5 or Tau-Cy5. The microglial activation rate was quantified as the percentage of CD68^+^ (white) area inside the microglial cell (stained with Iba1, green). In WT microglia, incubation with Tau-Cy5 increased the expression of CD68 compared to the PBS-Cy5 treatment at every time point tested (Fig. 1k). In contrast, in KO microglia, CD68 expression remained unchanged after the treatment with Tau-Cy5. In addition, the activation level of KO microglia cultured with PBS-Cy5 was abnormally high compared with WT microglia under same conditions. These data suggest that KO microglia showed alterations in their activation state that could be related to their impaired capacity to be further activated in the presence of Tau.

### The absence of CX3CR1 impairs the internalization of tau in vivo

**Fig. 2:**
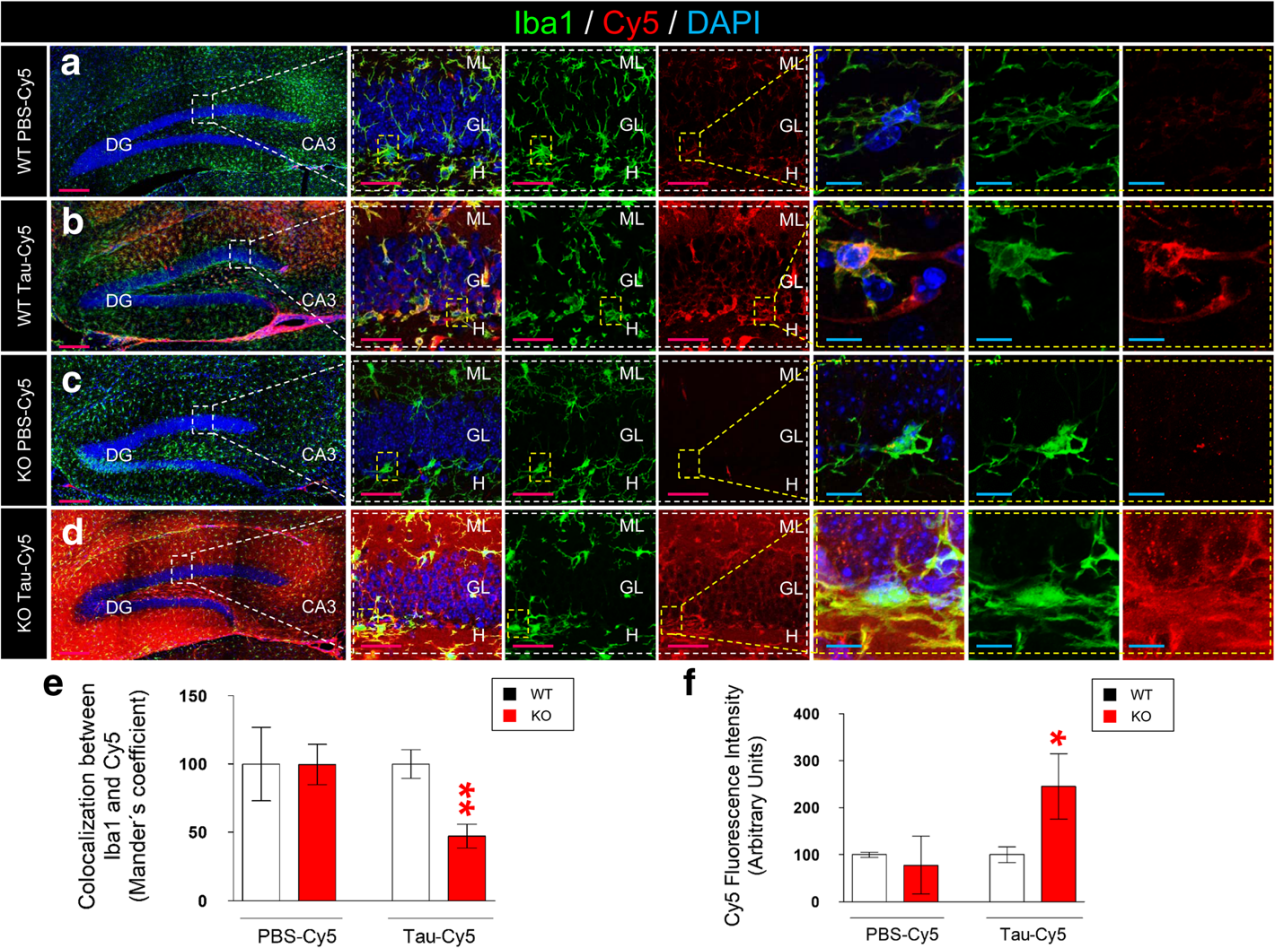
The absence of CX3CR1 leads to a decrease in Tau internalization by microglia in vivo*.* Representative images of the dentate gyrus (DG) of WT (**a**-**b**) and KO mice (**c**-**d**) stereotaxically injected with PBS-Cy5 or Tau-Cy5 and sacrificed one week later. Immunofluorescence images for Iba1 (*green*) and Cy5 (*red*) and high-power magnification are shown separately. **e** Colocalization between Iba1 and Cy5 in mice injected with PBS-Cy5 or Tau-Cy5 in the DG. Reduced colocalization between Cy5 and Iba1 can be observed in KO animals injected with Tau-Cy5 in comparison to WT mice that received the same injection. **f** Quantification of Cy5 fluorescence intensity in mice injected with PBS-Cy5 or Tau-Cy5. An increased level of Cy5 fluorescence intensity can be observed in KO mice injected with Tau-Cy5 in comparison to WT mice that received the same injection. Bars show means ± S.E. ∗*p* ≤ 0.05; ∗∗*p* ≤ 0.01; + 0.05 < *p* ≤ 0.1. Purple scale bar: 100 μm, Blue scale bar: 50 μm. GL, granule layer; H, hilus; ML, molecular layer; CA3, cornu ammonis region 3; DG, dentate gyrus

As our results suggested that Tau internalization is impaired in KO microglia in primary cultures, we next examined whether microglia take up Tau in vivo. With this aim, PBS-Cy5 or Tau-Cy5 were stereotaxically injected into the DG of WT and KO mice. After one week, the animals were sacrificed and immunohistochemical analyses were performed. After the injection of Tau-Cy5, the Cy5 signal (red) was observed in both WT (Fig. 2a-b) and KO (Fig. 2c-d) mice. Mander’s coefficient was used as a measurement of colocalization between the Cy5 signal and Iba1 staining (Fig. 2e). No differences in colocalization were found in PBS-Cy5-injected animals. Conversely, colocalization was reduced in Tau-Cy5-injected animals when KO and WT mice were compared (Fig. 2e). We next examined the levels of Tau remaining in the brain as a result of impaired internalization of Tau by microglia. For this propose, total Cy5 fluorescence was analyzed in the DG region of WT and KO mice injected with PBS-Cy5 or Tau-Cy5 (Fig. 2f). Although Cy5^+^ fluorescence was similar in WT and KO mice injected with PBS-Cy5, the Cy5 signal was markedly higher in the DG of KO mice injected with Tau-Cy5 than in that of WT animals receiving the same injection (Fig. 2f). Thus, we concluded that the absence of CX3CR1 impaired the internalization of Tau, thus possibly resulting in less efficient clearance of this protein by KO microglia. Interestingly, these results were consistent with those obtained in vitro.

In order to rule out the effect of early inflammatory response as a confounding factor, we injected the DG of a group of WT and KO animals with PBS-Cy5 or Tau-Cy5, as previously described, and sacrificed these animals 8 weeks later (Additional file 1). While there was no difference in Cy5 fluorescence intensity in animals injected with PBS-Cy5 (Additional file 1: Figure S1 a, c and e), the Cy5 signal was increased in Tau-Cy5-injected KO mice compared to Tau-Cy5- injected WT animals (Additional file 1: Figure S1 b, d and e). This observation indicates that, 8 weeks after injection, the levels of Tau were still significantly higher in the DG of KO mice. These data further support the notion that the impaired phagocytic capacity of microglia in KO animals has important long-term consequences for the brain micro-environment.

### Tau protein binds to CX3CR1

**Fig. 3:**
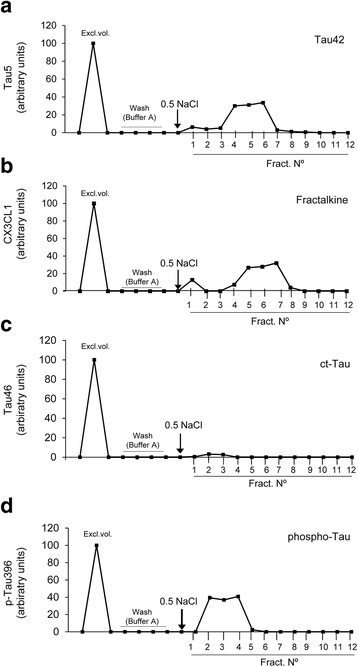
Tau binds to CX3CR1. Synthetic CX3CR1 was coupled to CNBr-activated Sepharose4B. Tau detected with anti-Tau5 (**a**), the positive control CX3CL1 (**b**), the negative control c-terminal Tau detected with anti-Tau46 (**c**), and Tau phosphorylated by GSK-3β detected with anti-pTau396 (**d**) were loaded on the column, as described in the Methods section. Each protein sample was used at a concentration of 1 mg/ml. Exclusion volume (excl. Vol.), aliquots corresponding to the column washed with buffer A, bound proteins eluted by addition of 0.5 M NaCl (marked with an *arrow*), and collected fractions (Fract. N°) are shown. Note that the profiles indicate that Tau, CX3CL1 and phospho-Tau were bound to the column, whereas the C-terminal fragment of Tau was not

Our results showed that CX3CR1 was involved in the internalization of Tau by microglia. In addition, the activation of microglia in response to Tau was attenuated in KO compared to WT microglia. Therefore, we addressed whether Tau serves as a ligand of CX3CR1 and whether it can bind to this receptor. To this end, we first performed affinity chromatography studies with the synthetic receptor CX3CR1 coupled to CN-sepharose (Fig. 3a-d). The exclusion volume of each peptide was collected, and the column was washed as described in Methods section. Next, 0.5 M NaCl in column buffer was used to elute the proteins bound to CX3CR1. Several 25-μl aliquots containing the bound proteins were collected, and the amount of protein was quantified by immunoblotting. Purified Tau, detected with a Tau5 antibody, was retained in the column (Fig. 3a). CX3CL1, the natural ligand of CX3CR1 used as a positive control for the experiment, was also retained in the column (Fig. 3b). CX3CL1 and Tau share 37% identity in some amino acid sequences (Additional file 1: Figure S2). These sequences have a high number of positively charged amino acids like K, R and H. This composition could explain why Tau and CX3CL1 bind to CX3CR1. In contrast, the C-terminal fragment of Tau holds negatively charge amino acids like D and E. Therefore, we used the C-terminal fragment of Tau as a negative control. This fragment, detected by the Tau46 antibody, was not retained in the column (Fig. 3c), thereby indicating that it was not able to bind to CX3CR1. Finally, given the pathologic relevance of phospho-Tau in AD, we examined whether it can bind to CX3CR1. Tau was phosphorylated by GSK-3β, as described in the Methods section. First, using western blot, we confirmed that Tau was phosphorylated after the enzymatic assay with GSK-3β (Additional file 1: Figure S3). We then performed the chromatography assay with phospho-Tau following the same protocol. Phospho-Tau, detected by the p-S396 antibody, was retained in the column (Fig. 3d). These results suggested that Tau bound to CX3CR1 regardless of its phosphorylation state. However, the profile shown in Fig. 3d suggested that the binding of phospho-Tau to CX3CR1 was weaker than that shown by Tau (Fig. 3a) since the former eluted earlier than the latter (fraction number 1 compared to fraction number 4).

**Fig. 4:**
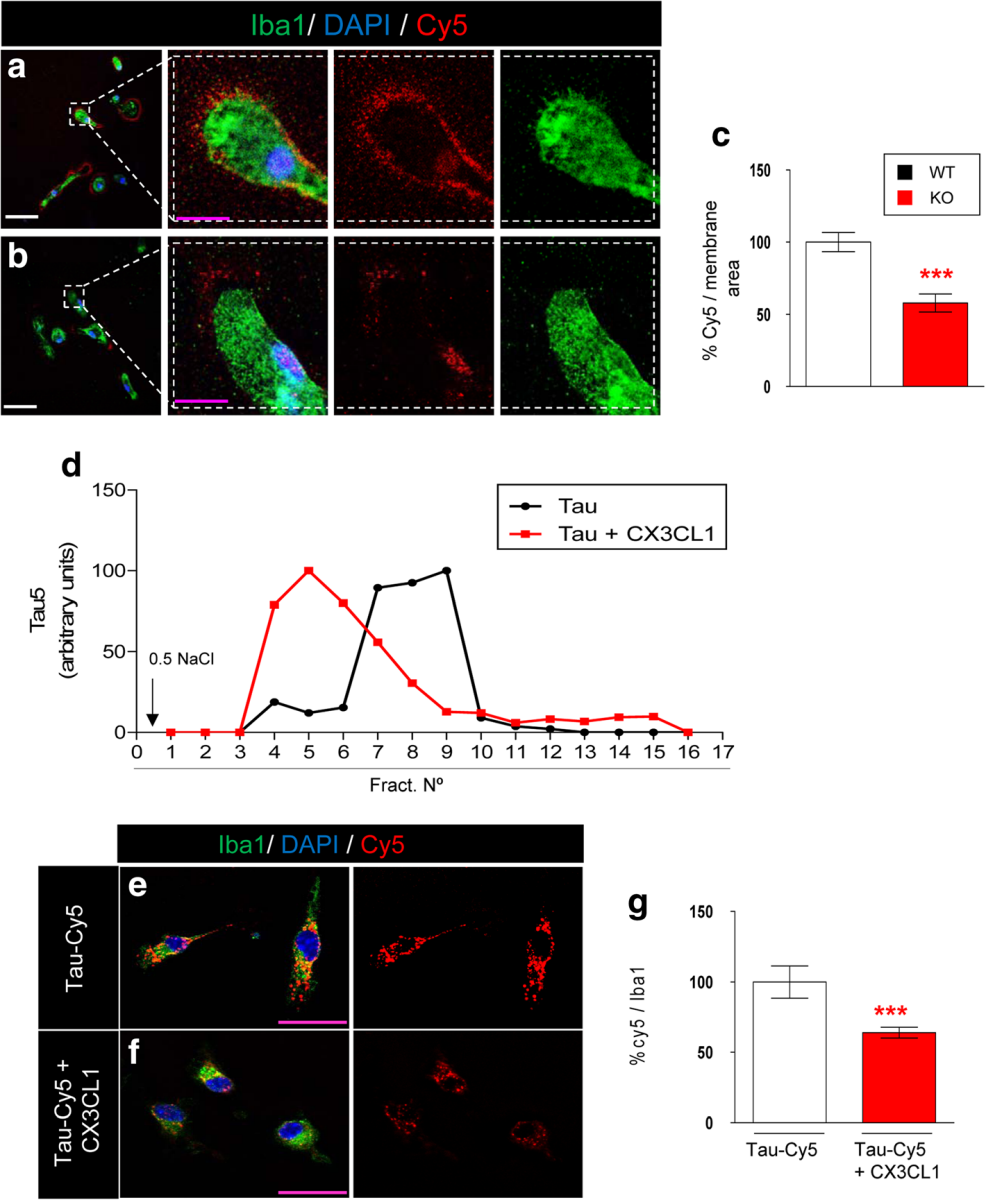
Tau competes with CX3CL1 for binding to CX3CR1. **a**-**b** Representative images of Tau-Cy5 attached to the surface of WT (**a**) and KO (**b**) microglia after treatment with Tau-Cy5 for 5 min at 4 °C. **c** Quantification of Cy5 fluorescence intensity on the microglial surface. In the absence of CX3CR1, the levels of Tau-Cy5 on the membrane of microglial cells are markedly reduced. **d** Tau profile in the competition assay between Tau and CX3CL1 detected with anti-Tau5. Note that the profiles indicate that the binding of Tau to the column was weaker in the presence of CX3CL1. **e**-**g** In vitro competition assay performed in primary microglial cell culture. Representative images and quantification of Cy5 fluorescence intensity (*red*) and Iba1 (*green*) in cells treated with Tau-Cy5 in the absence (**c**) or presence (**d**) of CX3CL1. As shown, Tau internalization is reduced in the presence of CX3CL1. Bars show means ± S.E. ∗∗∗*p* ≤ 0.001. White scale bar: 100 μm, purple scale bar: 50 μm

To determine how critical CX3CR1 was for the binding of Tau to microglia, microglia cultures from WT (Fig. 4a) and KO (Fig. 4b) mice were incubated with Tau-Cy5 at 4 °C for 5 min. The level of Tau on the microglial surface was measured (Fig. 4c). There was more Tau attached to the microglial surface in WT than in KO animals, which indicated that CX3CR1 also contributes to the binding of Tau to this surface.

### Tau competes with CX3CL1 for binding to CX3CR1

Given that CX3CL1 is the natural ligand of CX3CR1, we next studied whether CX3CL1 and Tau compete to bind CX3CR1. To this end, affinity chromatography was used to determine the amount of Tau retained in the column in the absence or presence of CX3CL1. The amount of Tau protein was quantified in the aliquots of eluted medium by immunoblotting. The binding of Tau to CX3CR1 was weaker in the presence of CX3CL1 (Fig. 4b). As shown, Tau eluted earlier in the presence of CX3CL1 (fractions 4–9) than in its absence (fractions 7–10).

Subsequently, in order to study whether the competition between Tau and CX3CL1 had an effect on Tau internalization, we addressed whether Tau internalization by microglia was affected by the presence of CX3CL1 in vitro. For this purpose, primary microglia cultures were treated with Tau-Cy5 in the absence or presence of CX3CL1 (Fig. 4c-d), and the internalization of Tau was measured by Cy5 fluorescence intensity (Fig. 4e). In the presence of CX3CL1, the internalization of Tau was reduced, as shown by the decreased Cy5^+^ signal (Fig. 4e). All together, these results reinforce the idea that CX3CR1 is involved in the internalization of Tau by microglia through a specific ligand-receptor binding mechanism.

### Phosphorylated tau and phagocytic phenotype of microglial cells in AD patients

**Fig. 5:**
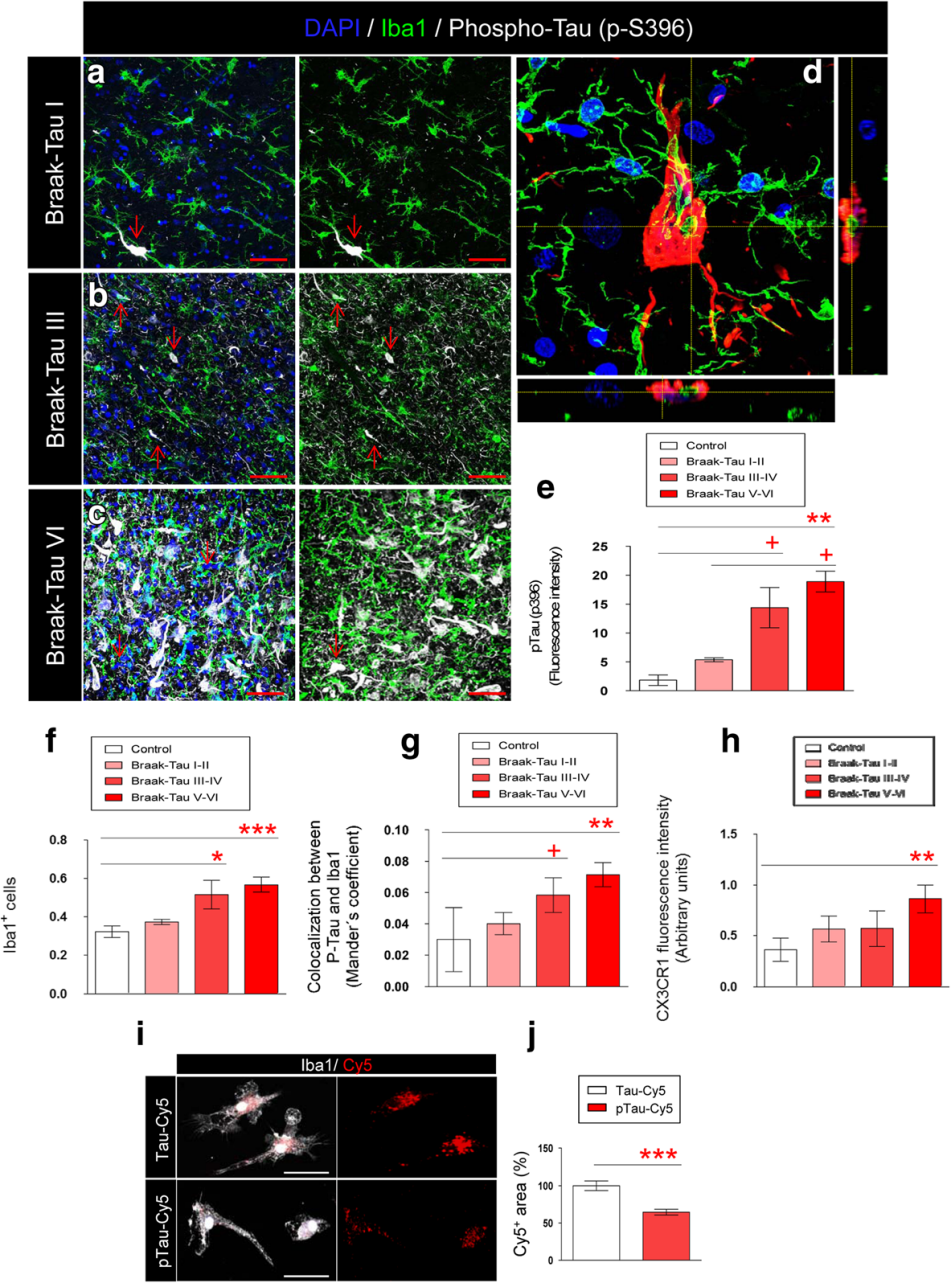
Tau phosphorylation, the number of microglial cells, colocalization between phosphorylated Tau and CX3CR1, and CX3CR1 expression increase in advanced stages of AD**.** Representative images of hippocampal tissue derived from AD patients showing microglia (Iba1, *green*), phospho-Tau (p-S396, *white*, *red arrow*) and DAPI (*blue*) staining at different stages of the disease: Braak-Tau I (**a**), Braak-Tau III (**b**) and Braak-Tau VI (**c**). Immunofluorescence images for Iba1- (*green*) and Tau-labeled structures (*white*, *red arrow*) are shown separately. **d** Representative high-power magnification image of microglia (*green*) and phospho-Tau (*red*) in Braak-Tau stage VI are shown. Orthogonal views are provided to highlight the colocalization between microglia and phospho-Tau. **e** Quantification of phospho-Tau fluorescence intensity present in the human hippocampus in AD. **f** Quantification of the number of microglia. **g** Colocalization between Iba1 and phospho-Tau at different stages of the disease. **h** Quantification of fluorescence intensity of CX3CR1 in the human hippocampus. (i-j) Representative images and quantification of the Cy5^+^ area relative to the internalization of Tau-Cy5 or phospho-Tau-Cy5 by microglia in vitro. Cell density was calculated as number of cells/μm^3^. Bars show means ± S.E. + *p* ≤ 0.05; ∗*p* ≤ 0.05; ∗∗ *p* ≤ 0.01 and ∗∗∗*p* ≤ 0.001. Red scale bar: 40 μm. White scale bar: 50 μm

In 1991, Braak and Braak proposed a neuropathological classification to differentiate Braak-Tau initial (I–II), intermediate (III–IV), and advanced (V–VI) stages of AD on the basis of the spread of neurofibrillary tangles within the brain [34]. However, there is limited quantitative information on the relationship between Braak-Tau staging and the phagocytic activity of microglia. To address this question, both the number of Iba1^+^ cells (green) and phospho-Tau staining (white, red arrows) were quantified in postmortem tissue derived from control subjects displaying a Braak-Tau stage equal to 0 and in postmortem tissue from AD patients at different Braak stages (Fig. 5a-d). Representative images of Braak-Tau I (Fig. 5a), Braak-Tau III (Fig. 5b), and Braak-Tau VI (Fig. 5c) and a high-power magnification image of Braak-Tau VI (Fig. 5d) were shown. First, phospho-Tau (p-S396) staining was quantified (Fig. 5e). Hippocampal phospho-tau staining was enhanced as the Braak-Tau stage increased (Fig. 5a-e). Next, the number of Iba1^+^ microglial cells (green) was quantified (Fig. 5f). Braak-Tau III-IV and Braak-Tau V-VI patients showed a greater number of cells compared to control subjects. In order to analyze the interaction between microglial cells and phospho-Tau in the brains of AD patients, colocalization between phospho-Tau and Iba1 was measured. The colocalization was found to be increased at Braak-Tau stages V-VI stages (Fig. 5g).

To study whether the expression of CX3CR1 is differentially regulated at the distinct stages of the disease, CX3CR1 staining was measured (Fig. 5h). CX3CR1 expression augmented progressively with increased Braak-Tau stage, thus indicating that the system is plastically regulated in the brains of AD patients. Although there was an increase in CX3CR1 with the advance of Braak-Tau stage, the amount of phospho-Tau was also increased. Given that this increase may reflect a failure of microglia to internalize phospho-Tau, we phosphorylated Tau-Cy5 with GSK-3β (Additional file 1: Figure S4). We then measured the internalization of phospho-Tau-Cy5 by microglial cells and compared it to that of the non-phosphorylated form (Tau-Cy5) (Fig. 5i-j) in vitro. Importantly, the uptake of phospho-Tau-Cy5 by microglia was lower than that of Tau-Cy5 under the same experimental conditions (Fig. 5j).

**Fig. 6:**
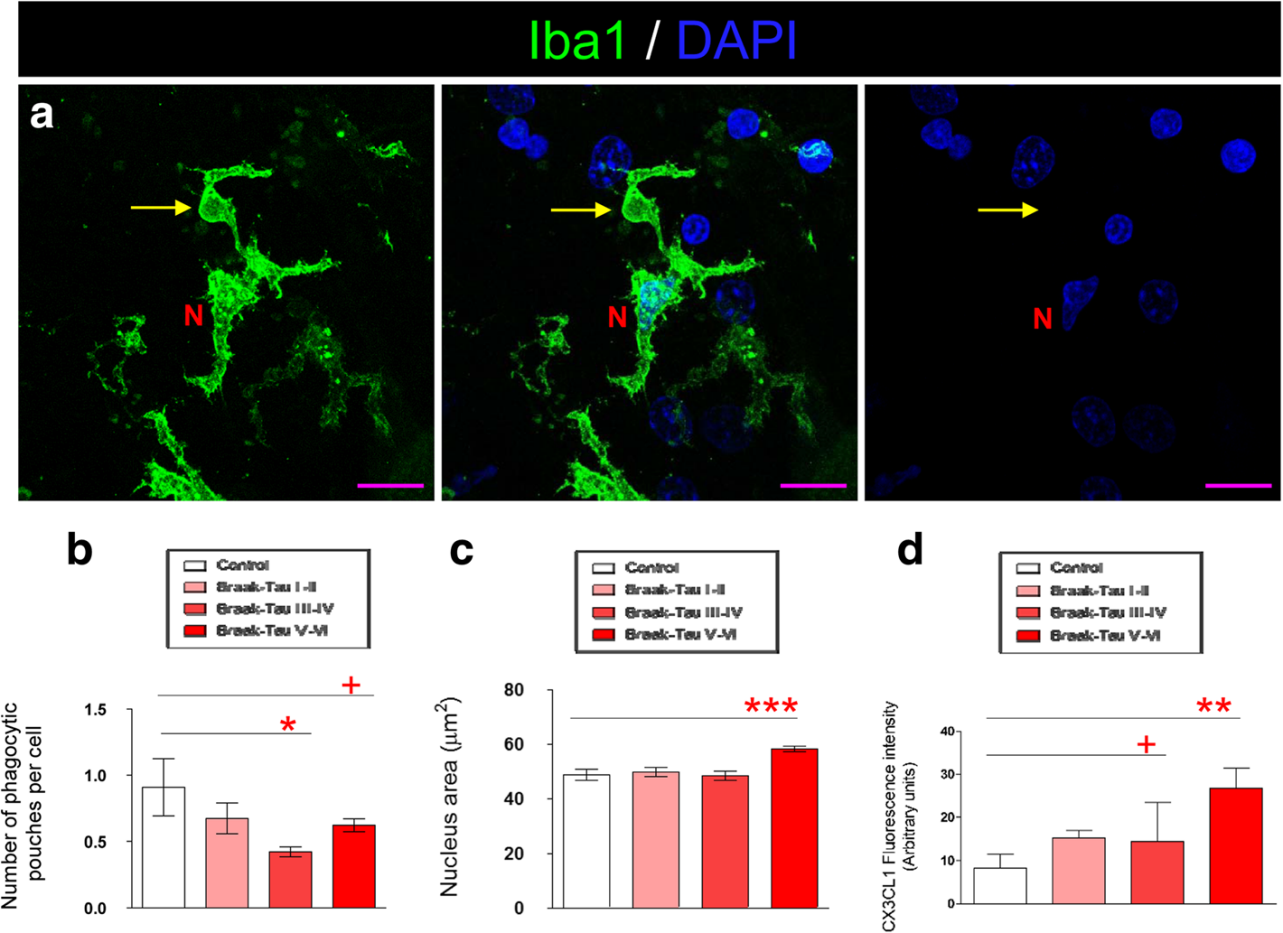
The impaired phagocytic capacity of microglia increases in advanced stages of AD. **a** Representative immunofluorescence images of phagocytic pouche (*yellow arrow*) in a microglial cell (Iba1, *green*) of human AD tissue. **b** Quantification of the number of phagocytic pouches per microglial cell. **c** Quantification of nucleus area of microglia in human tissue. **d** Quantification of CX3CL1 fluorescence intensity present in the human hippocampus in AD. Bars show means ± S.E. + *p* ≤ 0.05; ∗*p* ≤ 0.05; ∗∗ *p* ≤ 0.01 and ∗∗∗*p* ≤ 0.001. Purple scale bar: 40 μm. N, microglia nucleus (DAPI)

Taken together, these results (namely, the increased levels of phospho-Tau in the presence of an increased number of microglial cells) may point to impaired microglial phagocytosis at advanced Braak-Tau stages. In this regard, the phagocytic capacity of microglia was measured by quantifying the number of phagocytic pouches per cell. These pouches have been defined as a modification of microglial processes involved in phagocytosis in vivo [30, 31]. Representative images of these structures in the human brain are shown in Fig. 6a. Interestingly, a decrease in the number of phagocytic pouches was observed in Braak-Tau III-IV and V-VI patients (Fig. 6a). Furthermore, the area of the nuclei of microglial cells was measured as an indicator of their activation state. An increase in this parameter was found in Braak-Tau V-VI patients as compared to control subjects (Fig. 6c). In order to further explore the function of the CX3CL1/CX3CR1 axis in AD patients, the expression of CX3CL1 was measured in the DG of control subjects and AD patients at different Braak-Tau stages (Fig. 6d). CX3CL1 expression augmented progressively with increased Braak-Tau stage.

Taken together, these data further support our previous notion that an uncoupling of microglia activation-phagocytosis occurs in parallel with the exacerbated phosphorylation of Tau at advanced stages of the disease.

## Discussion

Microglia are resident immune cells that play a continuous surveillance role in the CNS [35]. Microglial phagocytosis is a crucial process both under physiological and pathological conditions [36]. In fact, genome-wide association studies have recently found genes associated with a higher probability of developing sporadic AD. Specifically, mutations in complement receptor 1 (CR1), the triggering receptor expressed on myeloid cells 2 (TREM2), and cluster of differentiation 33 (CD33) can result in reduced activity of the complement system and decreased phagocytosis [37–39]. These findings suggest that impaired microglial phagocytosis contributes to the progression of the disease. In line with this, it is known that the CX3CL1/CX3CR1 axis plays a central role in communication between microglia and neurons [10]. The present data show that microglia derived from KO mice show reduced in vitro phagocytosis of Tau compared to those from WT animals. Moreover, Tau binding to KO microglia is also reduced, thus suggesting that this receptor plays a key role not only in Tau internalization but also in Tau recognition and binding to the membrane.

In line with this, microglia from KO mice showed a reduced capacity in vivo to phagocytose Tau, as revealed by the decrease in the colocalization between the Cy5 signal and Iba1 staining. On the basis of our findings, we propose that the inefficient phagocytosis of Tau is detrimental for neighboring cells. Importantly, a similar hypothesis has recently been put forward for beta-amyloid [40].

Several studies, including our previous one [21], converge in the pivotal role played by microglia phagocytosis in the clearance of Tau and thus in preventing the spread of this protein and the progression of AD [2, 16, 22]. However, the mechanism of Tau clearance remains largely unknown. In neurodegenerative disorders, disruption of the CX3CL1-CX3CR1 axis has been described to have various effects. In studies conducted in KO mice, such disruption led to contradictory reports indicating either a protective effect [41] or worsening of the condition [42]. The present study is the first to show that Tau binds directly to CX3CR1, thereby contributing to its internalization by microglia. Tau shares positively charged amino acid residues with CX3CL1—a feature that may contribute to CX3CR1 binding. Additional site-directed mutagenesis experiments are now required to identify the specific amino acids involved in the binding of Tau to CX3CR1.

The analysis of brain tissue from AD patients revealed that an increase in the levels of phospho-Tau, the number of microglia, and the colocalization between phospho-Tau and Iba1 occurred in parallel as the disease advanced. It should be noted that in vivo experiments allow the study of colocalization but cannot ensure the internalization of this molecule into Iba1^+^ cells. We hypothesize that the amount of phospho-Tau adhered to the microglial surface increases at advanced stages of AD. However, given the higher amount of total phospho-Tau found in the tissue, it is not reasonable to assume that Tau phagocytosis is also increased, because it may be impaired at some point of the process. In fact, a decrease in the phagocytic activity of these cells was inferred by the reduction in the number of phagocytic pouches. Several authors have proposed that microglia malfunction with age [43, 44] and that this failure could be related to the development of AD. This “senescent” phenotype may include impaired phagocytic capacity. Our data support the notion that the uncoupling of microglial activation and phagocytosis occurs at later stages of AD. This is a phenomenon previously described in other neurodegenerative conditions [45]. Indeed, it has been proposed that neuron/microglial crosstalk is impaired in chronic neurodegenerative conditions [15]. This phenomenon may be the consequence of the chronic maintenance of a pro-inflammatory state throughout the advance of the disease that leads to system failure. Alternatively, it may constitute a neuroprotective mechanism aimed to counteract the excessive production of pro-inflammatory cytokines by microglia. Although unravelling the drivers of the decreased phagocytic capacity of microglia in AD is beyond the scope of the present study, we sought to determine the expression levels of CX3CR1 ligand, namely the neuronal cytokine CX3CL1, at various Braak-Tau stages. CX3CL1 expression was markedly increased at late stages of AD. This increase may act at two levels on the phagocytic capacity of microglia. On the one hand, CX3CL1 is an anti-inflammatory cytokine that retains microglial cells in a resting state [12]. However, importantly, on the other hand, our results demonstrate competition between Tau and CX3CL1 to bind CX3CR1*.* Whether or not the increase in CX3CL1 expression drives the impaired phagocytic capacity of microglia or whether these two phenomena merely occur in parallel in AD patients should be addressed by future studies. In addition, our in vitro data provide evidence that the internalization of phospho-Tau by microglial cells (triggered by its binding to CX3CR1) takes place in a less efficient manner than for the non-phosphorylated form of the protein.

Further studies are needed to elucidate whether the uncoupling of microglial activation and phagocytosis is due to exacerbated Tau phosphorylation driven by the increased expression of CX3CL1 or by alternative mechanisms. However, our in vitro data provide evidence of a reduced efficiency of microglia to internalize phospho-Tau and competition between Tau and the natural CX3CR1 ligand, CX3CL1, to bind this receptor.

These data reveal a novel mechanism of Tau internalization by microglial cells and further support the notion that the CX3CR1/CX3CL1 axis plays a critical role in the progression of AD.

## Conclusions

In this work we have demonstrated a novel mechanism of Tau internalization by microglia through the direct binding to CX3CR1. We have suggested an uncoupling of activation-phagocytosis of microglia which occurs in parallel with the exacerbated phosphorylation of Tau at advanced stages of the disease. Finally, CX3CR1/CX3CL1 axis plays a critical role in the progression of AD.

## Additional file

Additional file 1: This file contains supplementary Figures S1-S4. (PDF 847 kb)

## Abbreviations

AD: Alzheimer’s disease
BSA: Bovine serum albumin
CNS: central nervous system
CX3CL1: chemokine (C-X3-C motif) ligand 1
CX3CR1: CX3C chemokine receptor 1
Cy5: sulfoindocyanine Cy5 dye
DAPI: 4 ‘,6-diamino-2-fenilindol
EGFP: enhanced green fluorescent protein
KO: knock out
PB: phosphate buffer
PBS: phosphate saline buffer
PFA: paraformaldehyde
WT: wild type
