# Supplementary material for: Absence of CX3CR1 impairs the internalization of Tau by microglia
Source: Mol Neurodegener. 2017 Aug 15;12:59. doi: 10.1186/s13024-017-0200-1 (PMC5558740; doi:10.1186/s13024-017-0200-1)

Absence of CX3CR1 impairs the internalization of Tau by microglia

Marta Bolós[1,2,*,#]; María Llorens-Martín[1,2,3*]; Juan Ramón Perea[1,2]; Jerónimo Jurado-Arjona[1,2]; Alberto Rábano[4]; Félix Hernández[1,2]; Jesús Avila[1,2, #]

[1]Centro de Investigación Biomédica en Red de Enfermedades Neurodegenerativas (CIBERNED), Valderrebollo 5, 28041-Madrid, Spain.

[2]Centro de Biología Molecular "Severo Ochoa" CSIC-UAM, Nicolás Cabrera 1, 28049 Madrid, Spain.

[3]Department for Molecular Biology, Faculty of Sciences, Universidad Autónoma de Madrid (Spain).

[4]Neuropathology Department, CIEN Foundation, Madrid, Spain.

[*]These authors contributed equally to this work.

[#]corresponding authors: Jesús Avila and Marta Bolós, Centro de Biología Molecular 'Severo Ochoa', Universidad Autónoma de Madrid, C/ Nicolás Cabrera 1, 28049 Madrid, Spain. Tel.: +34-911964592. Fax: +34-911964420. E-mails: jesus.avila@csic.es/javila@cbm.csic.es and mbolos@cbm.csic.es.

Additional files

Additional file 1. The absence of CX3CR1 led to a decrease in Tau internalization by microglia of the dentate gyrus (DG) *in vivo* after 8 weeks. Representative images of WT (a-b) and KO mice (c-d) stereotaxically injected with PBS-Cy5 or Tau-Cy5 in the DG and sacrificed 8 weeks later. Immunofluorescence images for Iba1 (green) and Cy5 (red) and high-power magnification are shown separately. (e) Quantification of Cy5 fluorescence intensity in mice injected with PBS-Cy5 or Tau-Cy5. (f) Colocalization between Iba1 and Cy5 in mice injected with PBS-Cy5 or Tau-Cy5 in the DG. Bars show means ± S.E. $**p \leq 0.01$; $***p \leq 0.001$. Purple scale bar: 50 µm, Blue scale bar: 100 µm. GL, granule layer; H, hilus; ML, molecular layer; CA3, cornu ammonis region 3; DG, dentate gyrus.

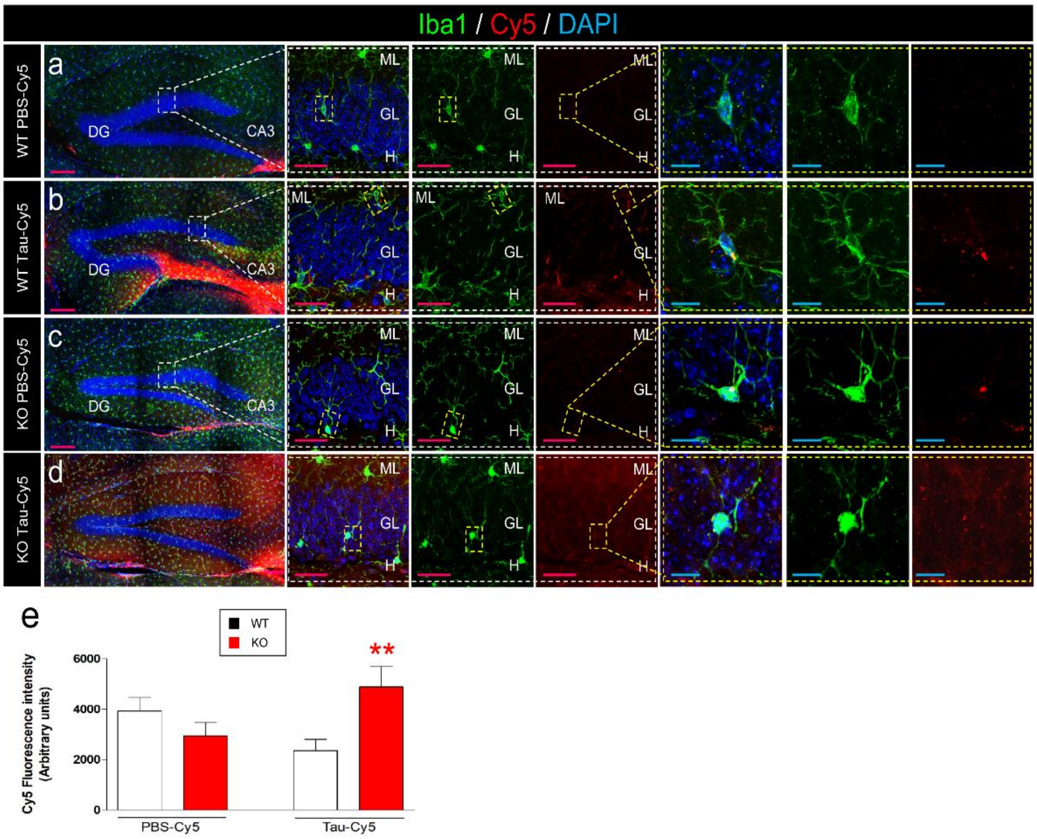

Additional file 2. Percentage of sequence identity between human Tau42 (a) and CX3CL1 (b) proteins. The sequences of human Tau42 and human CX3CL1 were obtained from NCBI. The amino acid sequences of human Tau42 (Reference Sequence: NP_005901.2) and human CX3CL1 (GenBank: CAG33707.1) were compared using the protein-protein BLAST tool (https://blast.ncbi.nlm.nih.gov). These proteins were found to share 37% sequence identity (c). Yellow: Human Tau identity sequences. Pink: CX3CL1 Tau identity sequences.

a)

Human Tau

MAEPRQEFEVMEDHAGTYGLGDRKDQGGYTMHQDQEGDTDAGLKESPLQTPTEDGSEEPGSETSDAKSTPTAEDVTAPLVDEGAPGKQAAA
QPHTEIPEGTTAEEAGIGDTPSLEDEAAGHVTQEPESGKVVQEGFLREPGPPGLSHQLMSGMPGAPLLPEGPREATRQPSGTGPEDTEGGRHA
PELLKHQLLGDLHQEGPPLKGAGGKERPGSKEEVDEDRDVDESSPQDSPPSKASPAQDGRPPQTAAREATSIPGFPAEGAIPLPVDFLSKVSTEIP
ASEPDGPSVGRAKGQDAPLEFTFHVEITPNVQKEQAHSEEHLGRAAFPGAPGEGPEARGPSLGEDTKEADLPEPSEKQPAAAPRGKPVSRVPQL
KARMVSKSKDGTGSDDKKAKTSTRSSAKTLKNRPCLSPKHPTPGSSDPLIQPSSPAVCPEPPSSPKYVSSVTSRTGSSGAKEMKLKGADGKTKIATPR
GAAPPGQKGQANATRIPAKTPPAPKTPPSSGEPPKSGDRSGYSSPGSPGTPGSRSRTPSLPTPPTREPKKVAVVRTPPKSPSSAKSRLQTAPVPMPDL
KNVKSKIGSTENLKHQPGGGKVQIINKKLDLSNVQSKCGSKDNIKHVPGGGSVQIVYKPVDLSKVTSKCGSLGNIHHKPGGGQVEVKSEKLDFKDRVQS
KIGSLDNITHVPGGGNKKIETHKLTFRENAKAKTDHGAEIVYKSPVVSGDTSPRHLSNVSSTGSIDMVDSPQLATLADEVSASLAKQGL

b)

Human CX3CL1

MAPISLSWLLRLATFCHLTVLLAGQHHGVTKCNITCSKMTSKIPVALLIHYQQNQASCGKRAIILETRQHRLFCADPKEQWVKDAMQHLDRQAALT
RNGGTFEKQIGEVKPRTTPAAGGMDESVVLEPEATGESSSPEPTPSSQEAQRALGTSPELPTGVTGSSGTRLPPTPKAQDGGPVGTELFRVPPVST
AATWQSSAPHQPGPSLWAEAKTSEAPSTQDPSTQASTASSPAPEENAPSEGQRVWGQGQSPRPENSLEREEMGPVPAHTDAFQDWGPGSMAH
VSVVPVSSEGTPSREPVASGSWTPKAEEPIHATMDPQRLGVLITPVPDAQAATRRQAVGLLAFLGLLFCLGVAMFTYQSLQGCPRKMAGEMAEGLRY
IPRSCGSNSYVLVPV

c)

Human Tau- Human CX3CL1 blast

```
VDLSKVTSKCGSLGNIHHK   human Tau
   SK+TSK      IH++   37% ident
ITCSKMTSKIPVALLIHYQ   human CX3CL1

SPGTPG-----SRSRTPSL--PTPPTREPKKVAVVRTPPKSPSSAKSR   human Tau
S GTP      S S TP    P   T +P+++ V+ TP     +A  R   37% ident
SEGTPSREPVASGSWTPKAEEPIHATMDPQRLGVLITPVPDAQAATRR   human CX3CL1
```

Additional file 3. Phosphorylation of Tau42 by GSK-3β. Western blot of phospho-Tau42 after enzymatic reaction with GSK3β. Anti-phospho-Tau (p-S396) antibody was used. C: Tau42 protein, control extract. 1: Tau42 phosphorylated by GSK3β following the methods described in the Methods section. 3: Tau42 without GSK3β (control of the enzymatic reaction).

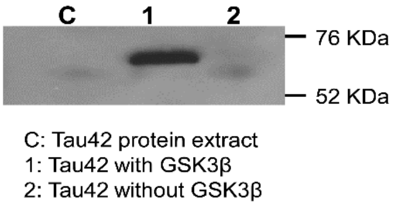

Additional file 4. Phosphorylation of Tau42-Cy5 by GSK-3β. Western blot of phospho-Tau42 after enzymatic reaction with GSK3β. Anti-phospho-Tau (p-S396) antibody was used. 1: Tau42-Cy5 phosphorylated by GSK3β following the method described in the Methods section. 2: Tau42-Cy5 without GSK3β (control of the enzymatic reaction).

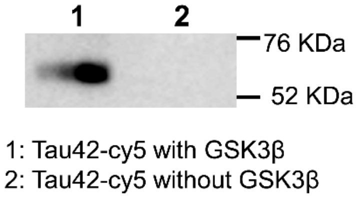

Supplement: Additional file 1: — This file contains supplementary Figures S1-S4. (PDF 847 kb) [file 13024_2017_200_MOESM1_ESM.pdf]
